# Supplementary material for: Empirical methods for controlling false positives and estimating confidence in ChIP-Seq peaks
Source: BMC Bioinformatics. 2008 Dec 5;9:523. doi: 10.1186/1471-2105-9-523 (PMC2628906; doi:10.1186/1471-2105-9-523)
Supplement: Additional File 1 — A variety of html documents from the USeq web site detailing the available applications, their best usage, output file type descriptions, command line menus, etc. [file 1471-2105-9-523-S1.zip › USeqUserGuides/JavaDoc.zip!/JavaDoc/gata/geneGlyphs/package-tree.html]

gata.geneGlyphs Class Hierarchy


|  |  |  |
| --- | --- | --- |
| **Overview** \| **Package** \| Class \| Use \| **Tree** \| **Deprecated** \| **Index** \| **Help** | |  |
| **PREV**   **NEXT** | **FRAMES**    **NO FRAMES**     **All Classes** |  |


---


## Hierarchy For Package gata.geneGlyphs

**Package Hierarchies:**: All Packages

---

## Class Hierarchy

- java.lang.Object
  - gata.geneGlyphs.**Annotation**- java.awt.Component (implements java.awt.image.ImageObserver, java.awt.MenuContainer, java.io.Serializable)
      - java.awt.Container
        - javax.swing.JComponent (implements java.io.Serializable)
          - javax.swing.JPanel (implements javax.accessibility.Accessible)
            - gata.geneGlyphs.**GlyphPanel**
      - gata.geneGlyphs.**GeneRepGlyph**- gata.geneGlyphs.**GenericGlyph**- gata.geneGlyphs.**ScaleBar**- gata.geneGlyphs.**TransGrpGlyph**

---


|  |  |  |
| --- | --- | --- |
| **Overview** \| **Package** \| Class \| Use \| **Tree** \| **Deprecated** \| **Index** \| **Help** | |  |
| **PREV**   **NEXT** | **FRAMES**    **NO FRAMES**     **All Classes** |  |


---
